# Previously unidentified duplicate registrations of clinical trials: an exploratory analysis of registry data worldwide

**DOI:** 10.1186/s13643-016-0283-8

**Published:** 2016-07-15

**Authors:** Gert van Valkenhoef, Russell F. Loane, Deborah A. Zarin

**Affiliations:** Department of Epidemiology, University of Groningen, University Medical Center Groningen, RB Groningen, PO Box 30.001, 9700 The Netherlands; National Library of Medicine, 8600 Rockville Pike, Bldg 38A, Rockville, 20894 MD USA

**Keywords:** Clinical trials, Trial registration, Duplicate registrations

## Abstract

**Background:**

Trial registries were established to combat publication bias by creating a comprehensive and unambiguous record of initiated clinical trials. However, the proliferation of registries and registration policies means that a single trial may be registered multiple times (i.e., “duplicates”). Because unidentified duplicates threaten our ability to identify trials unambiguously, we investigate to what degree duplicates have been identified across registries globally.

**Methods:**

We retrieved all records from the World Health Organization (WHO) International Clinical Trials Registry Platform (ICTRP) search portal and made a list of all records identified as duplicates by the ICTRP. To investigate how to discriminate duplicates from non-duplicates, we applied text-based similarity scoring to various registration fields of both ICTRP-identified duplicates and arbitrary pairs of trials. We then used the best similarity measure to identify the most similar pairs of records and manually assessed a random sample of pairs not identified as duplicates by the ICTRP to estimate the number of previously unidentified (or “hidden”) duplicates.

**Results:**

Two hundred eighty-five thousand unique records, or 271 thousand unique trials after accounting for known duplicates, were retrieved from the ICTRP portal in April 2015. We found that the title field best discriminated duplicates from non-duplicates. Out of 41 billion total pair-wise comparisons, we identified the 474,000 pairs of titles with the highest similarity scores (>0.5). After manually assessing a random sample of 434 pairs, we estimated that 45 % of all duplicate registrations currently go undetected and remain to be identified and confirmed as duplicates. Thus, the actual number of unique trials represented in this dataset is estimated to be approximately 258,000 (5 % less).

**Conclusions:**

The ICTRP portal does not currently enable the unambiguous identification of trials across registries. Further research is needed to identify and verify the duplicates that currently go undetected. Sponsors, registries, and the ICTRP should consider actions to ensure duplicate registrations are easily identifiable.

**Electronic supplementary material:**

The online version of this article (doi:10.1186/s13643-016-0283-8) contains supplementary material, which is available to authorized users.

## Background

Prospective registration of clinical trials, a scientific and ethical imperative, is required by various policies and laws throughout the world [1–4]. A key goal of trial registration is to enable identification of all conducted and ongoing trials relevant to a given topic. This goal can only be met if all relevant trials are registered, and the search system can accurately identify them, thereby avoiding both false positives and false negatives. Even when these two conditions are met, however, it is possible that duplicate registrations of the same study in one or more registries could interfere with the ability to account for all trials by making it impossible to determine how many trials actually exist [5]. Duplicates can occur when a trial sponsor purposefully registers their trial in more than one registry, or when different people associated with the trial (especially for multi-site, multi-national trials) register the trial in an uncoordinated fashion. This problem was recognized by the World Health Organization (WHO) Mexico statement [6], which called for “unambiguous identification” of each trial, and led to the establishment of the International Clinical Trials Registry Platform (ICTRP) by the WHO. The WHO also created the Universal Trial Number (UTN) scheme to facilitate the unambiguous identification of trials by allowing a unique number to be associated with a trial throughout its life cycle. Unambiguous identification is important both to prevent double (or triple) counting of evidence in systematic reviews and meta-analyses and to ensure that all registry records describing each relevant trial can be identified and retrieved. Registry data are also being used to study the clinical research enterprize itself [7–9], and duplicate registrations could skew the results of such studies. In addition, the unambiguous identification of trials is an important step on the way to enhancing the efficiency of systematic reviews using information technology [10].

If the different versions of the trial registration use the same trial identifier (e.g., in the secondary ID field) and/or use the same words to describe key trial attributes, then it can be easy to detect. However, this is frequently not the case. We have shown how some duplicates appear quite different (e.g., different titles, sponsors, and condition terms), and some non-duplicates appear to describe the same trial (e.g., same words for the title, sponsor, and condition) [5]. ClinicalTrials.gov, the largest registry, accepts trials from anywhere in the world. It takes steps to avoid and detect possible duplicates; when identified, only one version of the trial record is kept active. We are not aware of the processes used by other registries to either prevent or detect duplicate registrations.

In the following, we use *record* to refer to a trial as registered in a specific registry, and *variants* to refer to different versions of a record within that registry (see Table 1 for an example). Most registries list a single authoritative variant per record, but the European Union Clinical Trials Register (EUCTR) contains a variant of the protocol information for each member state in which the trial is registered. The ICTRP provides a consolidated search portal that currently lists over 285,000 records (over 320,000 variants) from 16 registries. Trials registered in more than one registry (or more than once in the same registry) would have more than one record in the ICTRP data. Although the ICTRP identifies some pairs (or triplicates) as representing the same trial (*known duplicates*), we have observed that they seem to not identify other apparent duplicates as representing the same trial (*hidden duplicates*). Systematic reviewers who use the ICTRP portal in an attempt to find a complete list of all trials relevant to their review would therefore unsure about how many unique trials truly exist. This poses an additional problem to be solved during the review, and it could affect the assessment of bias by inflating the number of trials that did not publish results. While these problems are familiar for those conducting systematic reviews based on the scientific literature, registries should be able to provide a solution. Unfortunately, the proliferation of trial registration policies and registries has created barriers to doing this, despite the existence of the ICTRP portal [5]. We conducted the present analysis to estimate the magnitude of this problem and to recommend ways to improve this situation.

**Table 1:** Illustrating how the terms trial, record, and variant are used

Trial	Record	Variant
SPD557-206 (Shire Plc)	EUCTR2011-004388-62	EUCTR2011-004388-62-BE
		EUCTR2011-004388-62-CZ
		EUCTR2011-004388-62-DE
		EUCTR2011-004388-62-HU
		EUCTR2011-004388-62-LV
		EUCTR2011-004388-62-PL
	NCT01472939	NCT01472939

A trial registered in both the European Union Clinical Trials Register (EUCTR) and ClinicalTrials.gov illustrates the distinction we make between a trial, a record, and a variant. A trial may have been registered in more than one registry, resulting in multiple records. An EUCTR record may have been registered in multiple member states, resulting in multiple variants of that record

## Methods

We retrieved the complete ICTRP dataset in XML format in April 2015 using a special arrangement set up by the WHO. The ICTRP dataset contains the 20 items of the WHO trial registration dataset [11], which includes the registration ID, secondary IDs, public and scientific titles, sponsors, contact information, conditions, interventions, outcomes, inclusion criteria, and several other fields. We removed HTML tags and clearly nonsensical secondary IDs and imported the data into a relational database.

The data did not contain the record groupings (i.e., the known duplicates) as displayed by the ICTRP search portal. To reproduce the record groupings, we matched the contents of the secondary ID field to record IDs by splitting the secondary ID into words (as separated by white space or one of the characters “,;.”) and looking for a matching record ID. For most registries, we looked for an exact match, while for EUCTR we allowed the EUCTR prefix and the country code (identifying a variant of the record) to be absent. The public EUCTR system is relatively new, but trials have been registered in the European Clinical Trials Database (EudraCT) for over a decade, so many references to records in the EUCTR lack the EUCTR prefix. We created groups of records based on the matched secondary IDs for all variants of each record and considered each record in a group to be a known duplicate of all other records in that group.

To estimate the number of hidden duplicates, we employed a two-stage strategy: scoring of pairs of records based on a text-based similarity measure followed by manual inspection of a random sample of highly scored pairs. The similarity measure developed for the first stage need not be truly predictive of a pair of records being duplicates; it only needs to reduce the number of candidate pairs sufficiently so that the second stage can be performed efficiently. The similarity scoring thus acts as a sieve that eliminates the vast majority of candidate pairs while only eliminating a small proportion of duplicates. This boosts the probability that a randomly selected pair are true duplicates and thus reduces the number of record pairs that have to be assessed manually.

To compute similarity scores, we normalized all the text (converted it to lowercase Latin characters, digits, and punctuation), tokenized it (separated it into individual words), and constructed a searchable index. When an EUCTR record had multiple variants, we used the variant that was registered first. For redacted records (i.e., public title “N/A” in EUCTR, or “[Trial of device that is not approved or cleared by the U.S. FDA]” in ClinicalTrials.gov), the similarity score was always set to zero. We considered the “concatenated” title (a concatenation of the public title, scientific title, and acronym, because these were not used consistently between registries), condition, intervention, outcome, and inclusion criteria fields [11]. Where these fields could occur multiple times, we simply concatenated their values. The mathematical details of our similarity scoring method can be found in Additional File 1 and are briefly summarized in the following. For each field, we assigned a weight to each word so that words that rarely occur in that field received high weight and words that occur frequently received low weight, using *inverse document frequency* [12, 13]. We also gave zero weight to stop words (e.g., “and” and “the”) and punctuation. To allow for optimizations (see Additional file 1), we did not assign greater weight to words that occurred more than once. However, we did consider the occurrence of the same word in different fields as two separate features. We used cosine similarity [14] to score the similarity of two records; a score of 0 means the records are completely different, and a score of 1 means the records contain exactly the same set of words in the same fields.

We first investigated the properties of our similarity scores to select the best fields for use in the final similarity scoring of record pairs. To do this, we computed similarity scores for all pair-wise comparisons between a random sample of 7000 records, as well as between all known duplicates. We computed scores for each field in isolation, as well as an overall score. We selected the field that resulted in the best discrimination between known duplicates and general pair-wise comparisons. Then, we computed and recorded similarity scores for all pairs of records that had a similarity of 0.5 or above on the selected field.

We drew random samples of pairs not known to be duplicates of each other, with title similarity scores in specific ranges, starting with 0.9−1.0. We judged whether each pair of records appeared to be duplicates of each other, using the criteria described below. We estimated the Binomial proportion and the corresponding Clopper-Pearson confidence interval [15] of pairs that were identified as duplicates. From the proportion we computed the estimated number of unidentified duplicates, which we compared with the number of known duplicates to compute the percentage of duplicates that remains hidden. We used the lower and upper bounds of the confidence interval for the proportion in the same way to compute a lower and upper bound for the percentage of duplicates that remains hidden. After obtaining a sufficiently accurate estimate for one interval, we would use the estimated percentage hidden to estimate the proportion of pairs that could be expected to be duplicates in the next interval (i.e., 0.8−0.9, then 0.7−0.8, etc.). We decided whether to continue the analysis based on the sample size that would be required to get an accurate estimate of the percentage of hidden duplicates in the next interval.

**Fig. 1:**
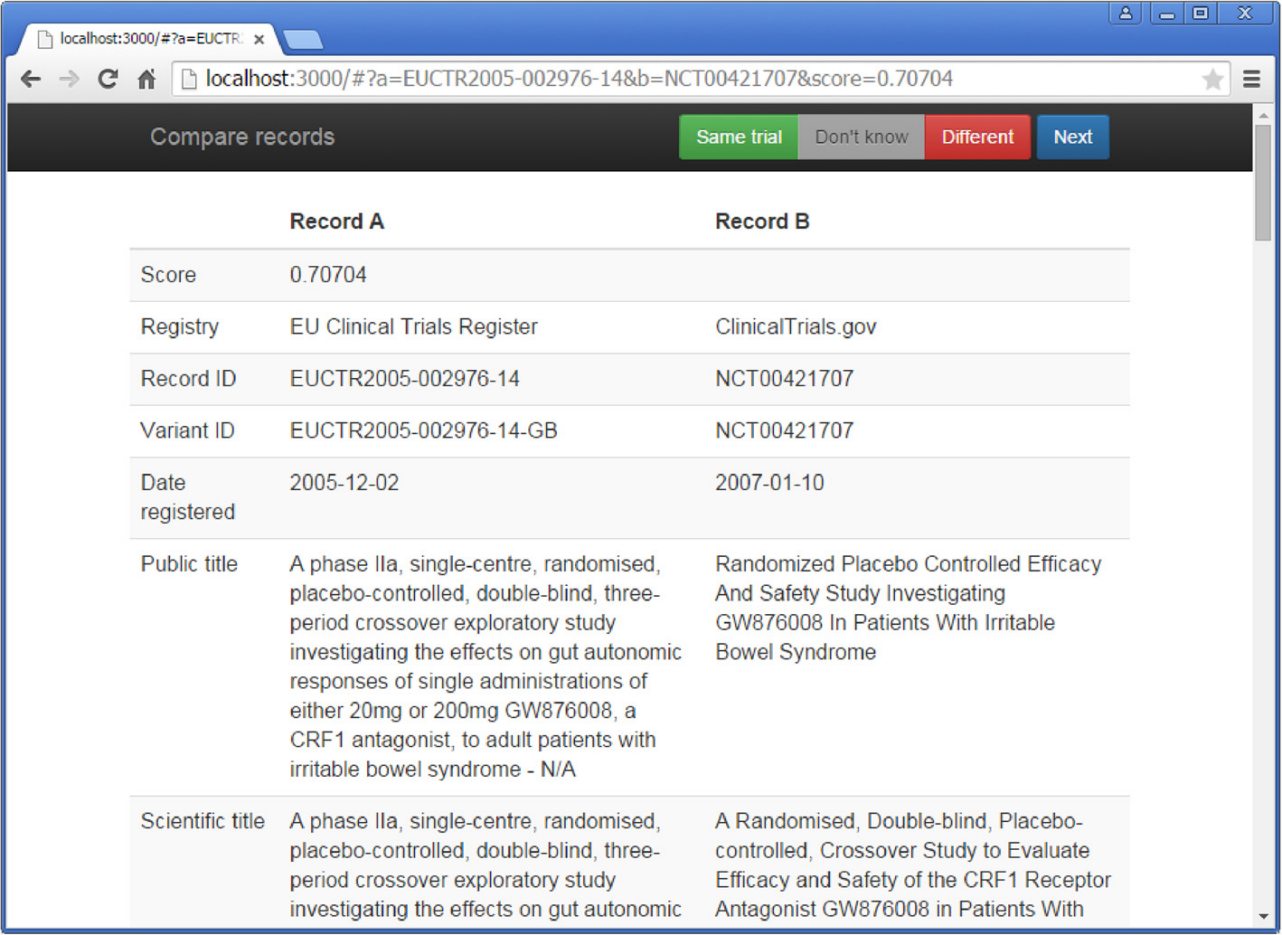
Record comparison user interface. Records were compared using a simple web application that shows two records side-by-side. The rater could use the “Same trial,” “Don’t know,” and “Different” buttons to indicate their judgment and proceed to the next pair by clicking “Next”. Mistakes could be corrected by simply clicking the correct button afterwards

Pairs of records were determined to be likely duplicates or non-duplicates using a web interface that showed two records side-by-side (Fig. 1). Rating was performed by the first author, who has research experience in meta-analysis and systematic review and detailed knowledge of registry systems and data. Difficult cases were discussed with co-authors and colleagues at ClinicalTrials.gov. The summary protocol information provided in the records are not sufficiently detailed to allow for the identification of duplicates with certainty. Therefore, we used a number of signals to determine the most likely answer. If the titles were nearly identical but contained clearly contradictory phrases (e.g., “fed conditions” versus “fasting conditions”), the trials were judged to be non-duplicates. If the trials shared a sponsor and had two identical secondary IDs that were not US federal grant numbers (which often apply to many different trials) and that were not likely to occur by chance (i.e., longer than five characters or digits), we judged the trials to be (hidden) duplicates. On the other hand, if the trials shared a sponsor and had different secondary IDs in the same format, we judged them to be different. Absent such cues, similarity of the titles, conditions, interventions, inclusion criteria, and outcomes were judged. As each registry has different requirements and quality assurance processes, the format and level of detail of these fields vary greatly between registries, so no objective criterion could be formulated. If these fields were sufficiently similar and the study start and end dates, sponsors, and investigators were not contradictory, the records were judged to be (hidden) duplicates. However, in case of doubt they would be judged non-duplicates. We followed ClinicalTrials.gov policy regarding follow-on studies: if an extension phase was part of the original protocol, it would be considered part of the same trial, but if an extension phase was set up post hoc under a new protocol, it was considered a new trial [16]. Such extensions entail additional data collection on some or all of the original trial’s population after it was planned to end and could involve alternative or additional interventions. We are not aware of the policies other registries have in this area but have observed that separately registered extension studies are also quite common in the EU Clinical Trials Register. Identifying extension studies and other interrelationships between studies might be valuable but is essentially a different research question that would shift the focus from a between registry problem to a within registry problem. Therefore, in our analysis, separately registered extension studies were not considered duplicates of the original trial.

Data processing programs were implemented in the Clojure programming language, and we imported the data into a MySQL database. Similarity scoring was implemented in a Java program using the ClinicalTrials.gov search engine “SE4” [17]. Records were compared using a small web application (shown in Fig. 1) using a Clojure server and an HTML/JavaScript frontend. Statistical analyses were carried out in the R statistical software. Full source code [18] is available from the Zenodo platform for sharing research outputs.

## Results

A total of 320,790 variants were retrieved from the WHO ICTRP portal, representing 285,177 unique records from 16 registries. The full dataset is available from Zenodo [19]. The five largest registries were as follows: ClinicalTrials.gov (187,554 records, 66 %), the EU Clinical Trials Register (25,179 records, 9 %), the Japanese Primary Registries Network (18,444 records, 6 %), ISRCTN (13,340 records, 5 %), and the Australian New Zealand Clinical Trials Registry (10,358 records, 4 %).

The dataset contained 387,264 secondary IDs, of which 55,206 could be matched to the registry ID of one of the records in the ICTRP dataset. In total, 26,201 records (9 %) had at least one known duplicate, reducing the assumed number of unique trials by 13,897 to 271,280. There were 10,932 groups of two records and 1372 groups of three or more records; the largest group contained ten records. Most duplicates occurred between registries, as only 55 groups of two or more records occurred within a single registry. However, the maximum number of registries in any group was five, and 111 groups had records in four or five registries.

**Fig. 2:**
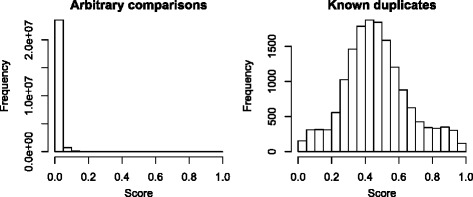
Comparing the similarity scores of arbitrary pair-wise comparisons to those of known duplicates. Histogram of the overall (combined) similarity scores of pair-wise comparisons between a random sample of 7000 records (*left*) compared to the similarity scores of known duplicates (*right*)

**Fig. 3:**
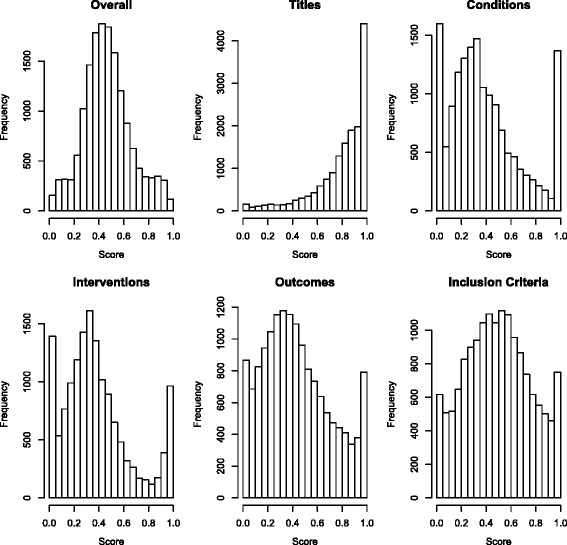
Distribution of similarity scores for known duplicates. Each panel is a histogram of the similarity scores for the population of known duplicates on one of the five considered fields or the overall (combined) score

The initial analysis showed that similarity scores between arbitrary pairs of records were highly concentrated near zero, regardless of the field that was chosen. Figure 2 illustrates this for the overall similarity scores for arbitrary pairs of records and contrasts it with overall scores for the known duplicates. Figure 3 shows the score distribution for the 15,805 pairs of known duplicates for the overall score and each of the five fields. Because the title field resulted in the best discrimination between duplicates and non-duplicates, it was used in all subsequent analyses. The median title similarity score for known duplicates was 0.86 (IQR 0.71–0.96), and 90 % of all known duplicates had a score exceeding 0.5.

We identified all pairs of records with a title similarity score of 0.5 or greater. There are 41 billion pair-wise comparisons between all records, of which 474,000 had a score of 0.5 or greater. Using an optimization described in Additional file 1, we needed to calculate only 206 million comparisons, which took 6 h on a desktop computer with an Intel Core i7-4790 Quad Core processor and 8 GB RAM.

**Fig. 4:**
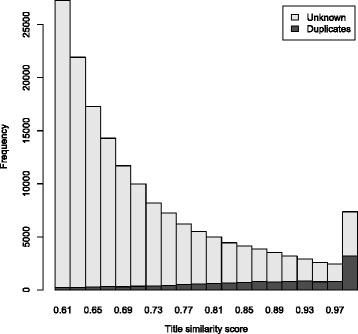
Known duplicates among highly similar records. Highly similar pairs (*light gray*) and the fraction that are known duplicates (*dark gray*). Pairs with similarity between 0.5 and 0.6 were identified but are not shown due to their large number (64 % of all pairs with a score over 0.5)

**Fig. 5:**
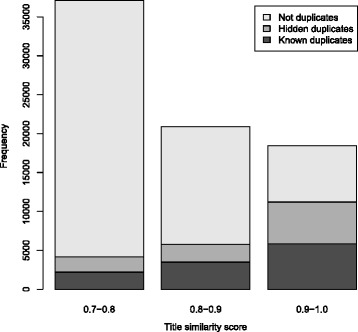
Estimated number of unknown duplicates. The number of unknown duplicates estimated by randomly sampling from the pairs of records that are not known to be duplicates. The investigated range of title similarity scores (0.7–1.0) contains 76 % of all known duplicates

The results are shown in Fig. 4: there are a large number of records with highly similar titles that are not known to be duplicates of each other. We estimated the proportion of those pairs that are in fact duplicates for the ranges 0.9−1.0, 0.8−0.9, and 0.7−0.8, covering 76 *%* of known duplicates. In total, 434 pairs were assessed, with the number assessed in each interval selected based on the width of the 95 % confidence interval for the percentage of duplicates that remain hidden. The results are shown in Table 2 and Fig. 5. For the score range 0.9−1.0, 43 % of assessed pairs were likely duplicates, for 0.8−0.9 this was 13 %, and for 0.7−0.8 it was 5.6 %. These numbers correspond to respectively 48, 39, and 47 % of duplicate registrations remaining unidentified. Overall, it appears that only about 55 *%* of duplicates have been identified in the ICTRP portal. In the 0.6−0.7 similarity score range, we expected only 1.1 *%* of sampled pairs to be duplicates. To bound that value between 0.5 and 2.6 *%* (corresponding to 25 and 65 *%* hidden duplicates, respectively) would require a sample size of 800 pairs. Therefore, we did not continue the analysis into the 0.6−0.7 range.

**Table 2:** The estimated number of unknown duplicates based on a random sample from each title similarity score range. Confidence intervals for the percentage of hidden duplicates based on the exact binomial confidence interval for the proportion of duplicates in the sample

Score range	D. in sample	D. known	D. unknown (est.)	% hidden
0.7<*x*≤0.8	7 / 125 (5.6 %)	2194	1957	47 (26–64)
0.8<*x*≤0.9	13 / 100 (13 %)	3489	2265	39 (26–51)
0.9<*x*≤1.0	89 / 209 (43 %)	5805	5393	48 (44–52)

## Discussion

Our analysis shows that the actual number of duplicates in the ICTRP dataset could be twice as high as the number currently identified as duplicates by the ICTRP. This would correspond to a reduction of the number of unique trials in the ICTRP dataset from 271,000 records currently to 258,000 actually unique studies (5 % less).

### Recommendations for systematic reviewers

Systematic reviewers cannot afford to ignore registry data when conducting systematic reviews [20], because registry data is key to determining how many studies have been done and how many have not published their results. Therefore, registries should be considered as part of the search strategy for any review. Reviewers will have to weigh the cost and benefit of using the ICTRP portal and/or individual registries, and future research should investigate the precision and recall of various strategies to aid such decisions.

Systematic reviewers need to be aware of the limitations of the ICTRP portal and the individual registries. In the ICTRP portal, any moderately large set of search results is likely to contain some duplicate records that have not been identified as such. Therefore, de-duplication remains necessary, even for trials retrieved from registries. Fortunately, the process is no different from identifying duplicate publications of the same trial. The ICTRP does not collect all data reported in the individual registries, so after the initial screening phase we recommend that the full record should be retrieved from the original registry. For example, ClinicalTrials.gov includes references to publications, which can help to link registry records to publications.

### Recommendations for sponsors

We suggest that sponsors internally designate a single registry record as their primary record, and refer to it consistently as a secondary ID on all other records. This will ensure proper grouping in the ICTRP search portal and will enable systematic reviewers and other researchers to easily identify duplicate registrations. Owing to the fact that the secondary ID field does not provide the context required to interpret other secondary IDs, linking all registry records to an internal protocol ID is not sufficient. Sponsors should focus their efforts on keeping the primary record up-to-date; it could serve as the source material for periodic updates to secondary records.

### Recommendations for registries

Registries could be more proactive in eliciting other registry IDs from sponsors, for example by targeted questions based on registered study locations (e.g., a drug trial with a site in the EU would suggest that a record exists in the EUCTR). Registries could also link to records in other registries based on duplicates identified by the ICTRP, similar to how ClinicalTrials.gov displays publications identified by PubMed [21]. Unfortunately, the ICTRP portal does not currently allow a trial’s identified duplicates to be retrieved. We also recommend that registries enact policies and quality assurance processes to improve the quality of published records. For example, issues such as nonsense secondary IDs and multiple values per field should be minimized. In addition, given that clinical trials are increasingly international [22, 23], it would be ideal if registries could allow sponsors to update their secondary records by downloading the primary one from another registry.

It is unfortunate that each EUCTR record has a (potentially large) number of variants. We hope that the EUCTR will start offering a single authoritative variant for each record to the ICTRP in the future. That would remove a layer of complexity for anyone using the ICTRP data.

### Recommendations to the ICTRP

We believe significant benefit to clinical research could be achieved by making the ICTRP dataset more readily available, for example by publishing electronic snapshots of the full dataset at regular intervals. If the data were publicly available, third parties could improve the data in various ways, e.g., by standardizing fields between registries and mapping free text to controlled vocabularies.

The ICTRP portal is currently only accessible through a web browser. If other computer systems could execute queries and retrieve specific records in XML format, other services that deal with clinical trials would benefit. For example, the primary registries could more easily link back to the ICTRP portal, or directly to duplicate records in other registries. The ICTRP portal could also function as a linking hub for journal articles that refer to registry IDs. The ICTRP should play a central role in the international registry system, but given the current state of affairs, it is sometimes ignored. Instead, major studies using registry data have focussed their efforts on using ClinicalTrials.gov [7–9, 24–27], even though its coverage of trials conducted worldwide is not as comprehensive as that of the ICTRP portal.

The ICTRP dataset lacks standardization in some areas; date formats are inconsistently used, for example. The ICTRP should work with registries to further standardize how fields are reported and should communicate to data consumers what standards have been established. Addressing the issues of duplicate registrations and divergent reporting will only become more challenging as the number of independent registries grows. Therefore, the ICTRP should aim to limit the number of new registries, and instead focus on increasing the use of existing registries.

### Reflections on the secondary ID field

The secondary ID field has proven to be useful in allowing some records to be grouped in the ICTRP search results. The ICTRP does this by matching secondary IDs to the registry IDs of known records. There are good reasons why it does not match other secondary IDs, however. The secondary ID field has been used to enter many different kinds of identifiers that may not uniquely identify a trial, and there is no information to indicate what kind of identifier has been entered. For example, grant numbers are commonly entered, and the most common grant number occurred on 262 separate trial records. Nonsense values such as “Version 1” and “Nil known” are also common. It is especially puzzling that occasionally multiple IDs are entered in a single secondary ID field, because all registries allow multiple secondary ID fields to be used. Matching secondary IDs alone is also risky because different sponsors may use overlapping identifier schemes, with the use of incrementing integers being the most common.

Despite these issues, it must be possible to extract some additional value from the secondary ID field. We performed two small experiments to illustrate this. First, we took all secondary IDs that matched the WHO UTN identifier format. Surprisingly, the ICTRP portal does not appear to group trials by UTN (for example, DRKS00005274 and NCT02139163 both have the UTN U1111-1147-8393, but are not grouped in the search results). In total, 2058 UTNs were found, which resulted in 151 matchings between trials, 56 of which were previously unknown. Second, we harvested a list of 6750 trial identifiers from the GlaxoSmithKline study register (http://www.gsk-clinicalstudyregister.com/) and matched these to secondary IDs on studies where GlaxoSmithKline was listed as a sponsor (we matched 123 variants of the GlaxoSmithKline name). We found 4270 matching secondary IDs, which would result in 423 newly identified duplicates and reduce the number of registered GlaxoSmithKline trials by 6 %. Based on these two experiments, we believe that a significant number of hidden duplicates could be identified by using similar strategies based on secondary IDs with external corroborating evidence. However, such work should include an assessment of the false positive rate.

### Limitations

Our work has a number of limitations. Most importantly, we provide a rough estimate of the number of hidden duplicates, but do not identify what those duplicates are; our similarity measure is not specific enough to allow efficient identification of all hidden duplicates. Our estimate of the number of duplicates depended on a somewhat subjective manual assessment of whether two records represented the same trial, which we attempted to fortify by setting clear criteria. The reliability of our estimates could be increased by using multiple raters, but given the exploratory nature of this study, we used a single rater only. We also did not consider that some of the duplicates identified by the ICTRP could be false positives. The similarity scores were calculated in a simple way, and more advanced methods (e.g., machine learning) could improve the ability to discriminate duplicates and non-duplicates, which would allow a more precise estimate. However, a more precise estimate would be of questionable value, and adopting more advanced methods would have greatly increased research effort and computation times. Advanced methods also have their own issues, such as the potential for over-fitting to the data and reduced transparency of the results. In estimating the number of hidden duplicates, we assumed that the probability of a duplicate being hidden does not depend on its similarity score. Our analysis of the most similar records (covering 76 % of known duplicates) did not suggest that such a dependency exists, but if it does, we may have underestimated the number of hidden duplicates. We cannot rule out that such confounding exists, for example because well-coordinated sponsors might be more likely to both register using similar titles and link their records using secondary IDs. In any case, our conclusion that a large proportion of duplicates remain undetected would remain valid, even if the actual proportion might turn out to be somewhat larger. Another limitation is that we used the first registered variant to represent EUCTR records. In rare cases, some variants are in languages other than English, or are simply incomplete (presumably because a more complete variant exists). Handling of EUCTR records could therefore be improved by employing a language identification technique to preferentially select English language variants, as well as by selecting the most complete variant (though defining “most complete” could prove a challenge).

Our study is also limited by the quality of the ICTRP dataset, which draws from 16 disparate registries. Reporting patterns and quality vary greatly between registries, and few fields have standardized values. For example, a number of different date formats is in use, and date formats differ both between registries and between the various date fields within registries. All of the registries have different policies regarding the specific information that goes into each field. For example, the EUCTR, which only includes drug trials, has very structured information on interventions, with labeled sub-fields for different compound names (brand, product, and generic), the pharmaceutical form, and the dosing regimen. By contrast, ClinicalTrials.gov, which includes studies of all intervention types, requires the specification of the intervention types (behavioral, drug, device, etc.) as well as textual descriptions of the interventions. On the other hand, ClinicalTrials.gov has quality assurance in place that encourages specific descriptions of outcome measures [16], so there tends to be more information on outcome measures in ClinicalTrials.gov than in other registries. This partly explains the poor similarity of known duplicates on the conditions, interventions, outcomes, and inclusion criterion fields (see Fig. 4).

## Conclusions

Our analysis shows that approximately 45 % of all duplicate registrations currently go undetected. A concerted effort by policy makers, registries, and trial sponsors is needed to ensure that the unambiguous identification of trials is possible. We also call for snapshots of the complete WHO ICTRP dataset to be made publicly available for use by independent research groups, so that this challenge and other research questions can be addressed.

## Additional file

Additional file 1 Similarity scoring method. A PDF document containing the mathematical details of our similarity scoring method. (PDF 125 kb)
