# Supplementary material for: Previously unidentified duplicate registrations of clinical trials: an exploratory analysis of registry data worldwide
Source: Syst Rev. 2016 Jul 15;5:116. doi: 10.1186/s13643-016-0283-8 (PMC4946209; doi:10.1186/s13643-016-0283-8)
Supplement: Additional file 1 — Similarity scoring method. A PDF document containing the mathematical details of our similarity scoring method. (PDF 125 kb) [file 13643_2016_283_MOESM1_ESM.pdf]

Additional file 1: Similarity Scoring Method

We used the term frequency-inverse document frequency (tf-idf) framework to create feature vectors from the normalized text describing each document (registry record), taking into account the field (e.g. title, outcomes, etc.) in which each token occurred. We call the set of all documents D, and let each document $d \in D$ be described by a number of fields, e.g. d_j is the value of field j. The weight assigned to each token is then:

$$f(t, d, j) = [t \in d_j] \log \frac{|D|}{|\{x \in D : t \in x_j\}|} ,$$

where $[t \in d_j]$ represents the binary "frequency" of the term t in field j of document d: 1 if the term occurs and 0 otherwise. Term frequency is weighted by the inverse document frequency: rarer terms are given higher weight. An exception is made for stop words (e.g. "and" and "the") and punctuation, which are always assigned weight zero. We let each document be described by a (very long) vector of these scores $f(t, d, j)$, in a consistent order. Then, if the vector $\boldsymbol{v}$ describes one document, and the vector $\boldsymbol{w}$ another, we use cosine similarity to judge their similarity:

$$\cos(\boldsymbol{v}, \boldsymbol{w}) = \frac{\boldsymbol{v} \cdot \boldsymbol{w}}{\|\boldsymbol{v}\| \|\boldsymbol{w}\|}$$

This is the cosine of the angle between the two vectors, and as such takes values in $[0, 1]$ with 0 meaning the vectors are completely perpendicular, and 1 meaning they are parallel.

To reduce the number of comparisons that needed to be computed, we constructed a minimal set of words so that at least one of them would need to occur in a second record for their similarity to exceed a threshold θ:

Theorem 1. *Take vectors $\boldsymbol{v}$ and $\boldsymbol{w}$ for which $\|\boldsymbol{v}\| \leq \|\boldsymbol{w}\|$, and either $v_i = w_i$, or $v_i = 0$, or $w_i = 0$ (for all i). Let I be an index set of features such that $\frac{1}{\|\boldsymbol{v}\|^2} \sum_{i \in I} v_i^2 > 1 - \theta$. If $\sum_{i \in I} v_i w_i = 0$, then cos$(\boldsymbol{v}, \boldsymbol{w}) < \theta$.*

Proof.

$$\begin{aligned} cos(\boldsymbol{v}, \boldsymbol{w}) &= \frac{\boldsymbol{v} \cdot \boldsymbol{w}}{\|\boldsymbol{v}\| \|\boldsymbol{w}\|} \leq \frac{1}{\|\boldsymbol{v}\|^2} (\boldsymbol{v} \cdot \boldsymbol{w}) \\ &= \frac{1}{\|\boldsymbol{v}\|^2} \sum_{i \notin I} v_i w_i \leq \frac{1}{\|\boldsymbol{v}\|^2} \sum_{i \notin I} v_i^2 \\ &= 1 - \frac{1}{\|\boldsymbol{v}\|^2} \sum_{i \in I} v_i^2 < \theta \end{aligned}$$

□

Using the theorem, we can select a limited set of features of one document (corresponding to a $1 - \theta$ fraction of the total squared weight), and if another document does not share any of those features, their similarity is guaranteed to be below θ (technically, this only works if $\|\boldsymbol{v}\| \leq \|\boldsymbol{w}\|$, but if not we can just swap the roles of the two documents). Therefore, for each document we selected the highest weight features such that their squared weight just exceeds $1-\theta$, and performed an OR search for the corresponding terms to find potentially similar documents. The comparison threshold was set based on both the distribution of similarity scores for known duplicates and on estimates of the computation time needed (which were based on small trial runs).
